# Statistical process monitoring creates a hemodynamic trajectory map after pediatric cardiac surgery: A case study of the arterial switch operation

**DOI:** 10.1002/btm2.10679

**Published:** 2024-05-15

**Authors:** Daniel P. Howsmon, Matthew F. Mikulski, Nikhil Kabra, Joyce Northrup, Daniel Stromberg, Charles D. Fraser, Carlos M. Mery, Richard P. Lion

**Affiliations:** ^1^ Department of Chemical and Biomolecular Engineering Tulane University New Orleans Louisiana USA; ^2^ Texas Center for Pediatric and Congenital Heart Disease University of Texas Health Austin and Dell Children's Medical Center Austin Texas USA; ^3^ Department of Surgery and Perioperative Care, Dell Medical School The University of Texas at Austin Austin Texas USA; ^4^ Department of Pediatrics, Dell Medical School The University of Texas at Austin Austin Texas USA; ^5^ Chandra Department of Electrical and Computer Engineering the University of Texas at Austin Austin Texas USA

**Keywords:** computational modeling, congenital heart disease, medical devices, postoperative monitoring, statistical process monitoring

## Abstract

Postoperative critical care management of congenital heart disease patients requires prompt intervention when the patient deviates significantly from clinician‐determined vital sign and hemodynamic goals. Current monitoring systems only allow for static thresholds to be set on individual variables, despite the expectations that these signals change as the patient recovers and that variables interact. To address this incongruency, we have employed statistical process monitoring (SPM) techniques originally developed to monitor batch industrial processes to monitor high‐frequency vital sign and hemodynamic data to establish multivariate trajectory maps for patients with d‐transposition of the great arteries following the arterial switch operation. In addition to providing multivariate trajectory maps, the multivariate control charts produced by the SPM framework allow for assessment of adherence to the desired trajectory at each time point as the data is collected. Control charts based on slow feature analysis were compared with those based on principal component analysis. Alarms generated by the multivariate control charts are discussed in the context of the available clinical documentation.

**Translational Impact Statement**

This article employs statistical process monitoring (SPM) techniques, originally developed to monitor batch industrial processes, to monitor patients with congenital heart disease following surgery. SPM provides a framework closely aligned with clinical decision‐making since it incorporates interactions between variables, assesses how these variables change over time, compares the current patient to historical data from previous successful patients, and summarizes observations in one or two metrics. This retrospective study paves the way for prospective use of multivariate control charts by care teams in the pediatric cardiac intensive care unit.

**Abbreviations**
ABP: arterial blood pressure
ASO: arterial switch operation
BPM: beats per minute
CEN: clinical event note
CHD: congenital heart defect
dTGA: dextro‐transposition of the great arteries
HFDC: high‐frequency data capture
HR: heart rate
PCICU: pediatric cardiac intensive care unit
PCA: principal component analysis
PN: progress note
SFA: slow feature analysis
SPE: squared prediction error
SpO_2_
: pulse oximetry
SMD: standard monitoring device
SPM: statistical process monitoring
SVD: singular value decomposition

## 1 INTRODUCTION

Congenital heart defects (CHDs) are the result of improper development of the heart or blood vessels in utero and are the most common birth defect worldwide,^1^ occurring in 0.8%–1.2% of the population.^2^, ^3^ Approximately one‐quarter are considered “critical CHDs” that require surgery shortly after birth.^1^ Advances in surgical and medical technology have drastically lowered patient mortality from 14% in 1982 to <3% in 2019, allowing a shift in focus to improving morbidity and long‐term outcomes.^4^, ^5^, ^6^ However, the period immediately following surgery continues to be a high‐risk period for this population, with half of the cardiac arrests experienced by this population occurring within 48 h of postsurgical admission to the pediatric cardiac intensive care unit (PCICU)^7^, ^8^, ^9^ and 30‐day survival used as a benchmark for hospital performance.^10^, ^11^ Management of the postoperative recovery process is highly lesion specific and impacted by the patient's age and size at the time of operation as well as the hospital volume and experience.^10^ Furthermore, there is no established postoperative hemodynamic trajectory for any individual CHD surgery that could provide clinicians with crucial information regarding patient decompensation before it becomes severe.

CHD patients are typically cared for in dedicated PCICUs by a team of specialized pediatric nurses and physicians, with standard of care including continuous monitoring of more than 10 physiologic vital signs, often on multiple devices at each patient bedside.^12^ These standard monitoring devices (SMDs) allow physicians to set static thresholds on each vital sign and generate alarms if these univariate thresholds are exceeded. However, the unique physiologies of CHD patients and lack of established baselines^13^ makes these univariate alarms significantly less reliable than in the general population. Moreover, the static thresholds on SMDs do not account for the dynamics of a normal recovery process. Additionally, the data from SMDs is generally not readily accessible to the clinical team for manipulation or long‐term storage, restricting the analysis of this data to real‐time interpretation by the clinician. Thus, even though high‐frequency waveforms continuously appear on the bedside monitors, monitoring of vital signs and hemodynamics in PCICUs is largely restricted to real‐time visualization/interpretation and manually recording these observations on an hourly basis.^14^ This recording process is corrupted with high rates of missing or erroneous entries and provides an incomplete picture of the variability of these measurements over time. Due to clinical workload demands, these manually recorded entries are typically not entered in real‐time, with reported delays of 40 min from vital sign occurrence to entry in the electronic medical record,^15^ further complicating the current generation of early warning systems based on this low‐frequency data.

There has been growing adoption of high‐frequency data capture (HFDC) systems that collate data from a variety of monitoring systems into a single warehouse, allowing clinicians to visualize both raw data alongside some rudimentary derived metrics (e.g., electrocardiography traces along with heart rate mean and variability). These HFDC systems also allow the clinical team to visually compare current vital signs and hemodynamics with those recorded earlier in the patient's course of care, allowing unprecedented insight into the distribution and trajectories of vital signs. However, as noted by Goldsmith et al.,^16^ “for the inexperienced, fatigued, distracted, or unaware clinician, this raw data—even displayed in a cognitively digestible format—may be too complex to accommodate the throughput of human processors.” Providing improved visualizations and real‐time analytics may help fully utilize this high‐frequency data and alert clinicians only when there is substantial deviation from expected or allowable trajectories.

Data from HFDC systems have begun to be used by the cardiovascular research community to provide more informative analyses and alerts. Data‐driven approaches have been used to predict cardiorespiratory deterioration events in single‐ventricle patients during the interstage period^17^ and mechanistic strategies were used to estimate the probability of inadequate oxygen delivery following neonatal cardiac surgery.^18^ These approaches can summarize multivariate measurements, including those of different frequencies and levels of fidelity, into a single score that the clinician can use for assessment. However, it is not expected that patients instantaneously reach homeostasis after congenital heart surgery, and methods that can recognize normal baseline dynamics associated with recovery processes are warranted. Clinicians have begun to use “trajectory maps” to track the typical dynamics of groups of patients. Trajectory maps for a vasoactive inotrope score in critically ill children with shock were clustered into groups and linked to mortality outcomes.^19^ A similar strategy was used to cluster intracranial pressure trajectories following traumatic brain injury and link to adverse clinical outcomes.^20^ These univariate maps uncover patterns in the underlying recovery processes but require the entire time course to be collected before clustering into a group and do not provide a measure of adherence to the trajectory map, so clinicians are not given the opportunity to intervene and possibly shift the patient into a more desirable trajectory.

Statistical process monitoring (SPM) provides a framework for assessing adherence of a given patient in real‐time to a multivariate trajectory map of historical data from previous patients with similar disease processes. Univariate SPM methods have been used previously in cardiology to monitor health recovery processes,^21^ assess clinician/hospital performance on morbidity and mortality outcomes,^22^, ^23^, ^24^ and assess the effects of quality improvements.^25^, ^26^ In industrial batch process monitoring, more sophisticated SPM techniques have been developed to handle multivariate, nonstationary, and nonlinear time‐series and alert plant operators when batch dynamics deviate substantially from historical trajectories of good quality batches.^27^, ^28^, ^29^, ^30^ This is achieved with the use of multivariate control charts that compare the current trajectory to a history of successful trajectories. Analogously, this article employs multivariate SPM techniques to alert clinicians when vital sign and hemodynamic trajectories deviate substantially from historical data on patients with favorable clinical outcomes.

Latent variable (LV) techniques are a group of data‐driven methods that seek to reduce the dimensionality of the original data and locate particularly informative directions or manifolds in multivariate space. The most widely used technique is principal component analysis (PCA), which extracts LVs according to the amount of variability they can explain. Dynamic information is typically incorporated by simply appending the data matrix with time‐lagged measurements.^31^ Slow feature analysis (SFA) is a newer LV technique that makes more explicit use of dynamic information by performing PCA on the covariance matrix of first derivatives of the signals (after normalization and sphering).^32^ This organization of LVs by slowness allows for the separation of rapid faults from slower operating point deviations.^27^, ^31^

**FIGURE 1:**
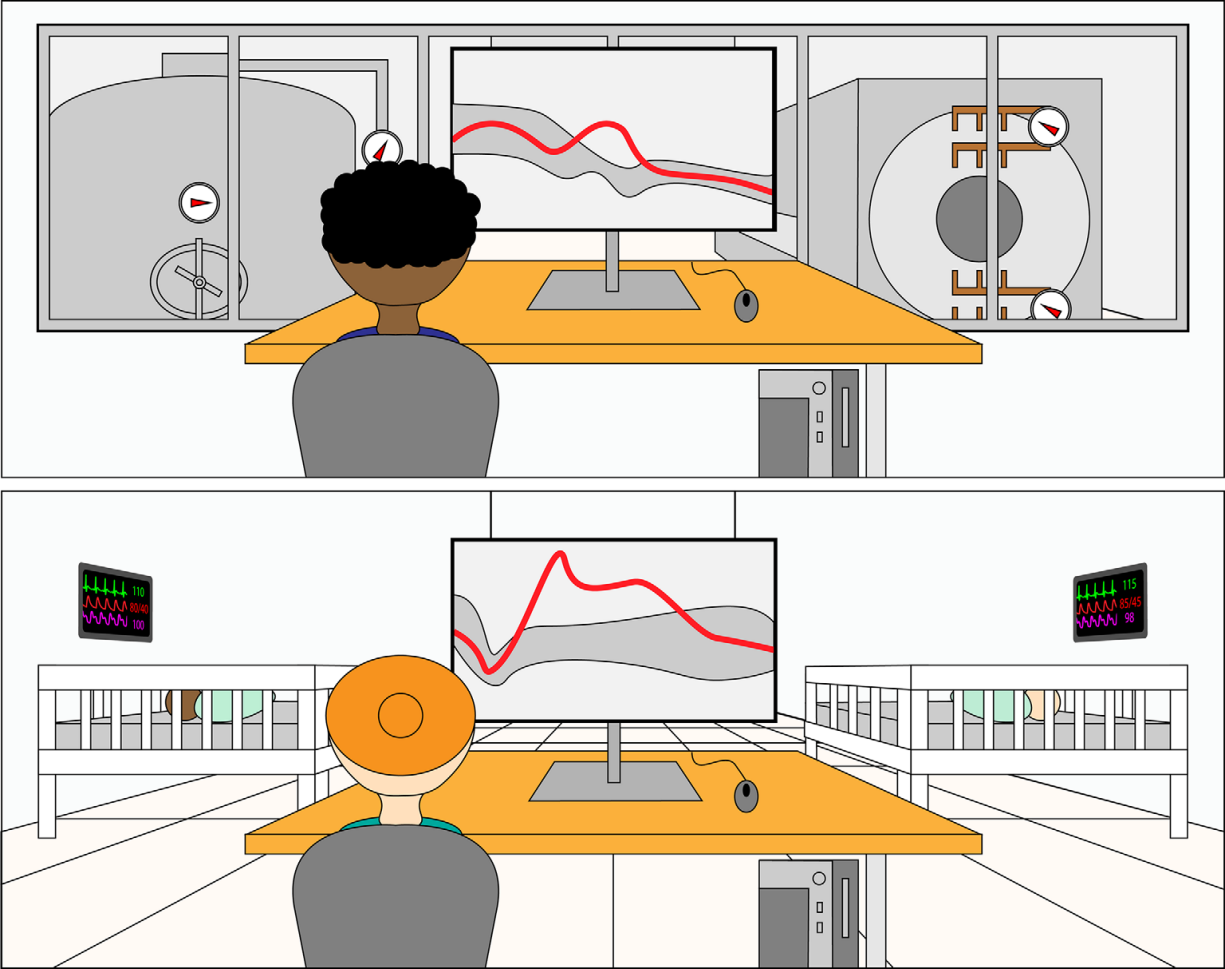
Illustration of the parallels between monitoring batch chemical processes and monitoring patient recovery.

This article leverages the SPM framework to make the connection between monitoring patients who are recovering from the same surgical procedure and monitoring batch processes (Figure 1). Specifically, LV techniques are used herein to model multivariate hemodynamic trajectories and SPM control charts are developed for monitoring pediatric patients with dextro‐transposition of the great arteries (d‐TGA), where the anomalous position of the pulmonary artery and aorta create largely independent pulmonary and systemic circulation systems. This defect causes significant cyanosis and shock within days of life if untreated.^33^ The standard of care for d‐TGA is surgical management via the arterial switch operation (ASO), which consists of redirecting pulmonary, systemic, and coronary blood flow in a single operation.^34^, ^35^, ^36^ The ASO is one of the most technically challenging procedures performed for CHD defects and has been considered an “index case” for measuring surgical/program outcomes and performance for more than a decade.^10^, ^37^, ^38^ This surgery was used as an initial case study to minimize confounding variables since patients undergoing ASO are relatively homogeneous with minimal variation in anatomic diagnosis, baseline patient demographics, and surgical approach at our institution.^34^, ^39^ Our postoperative monitoring strategy for the ASO removes outliers from individual time series, normalizes data to the baseline trajectory, constructs and compares LV models, and generates multivariate control charts to measure adherence to the expected trajectory. A discussion follows about the translation of SPM techniques to clinical decision support systems in the PCICU.

## 2 RESULTS

### 2.1 Cohort characteristics

**FIGURE 2:**
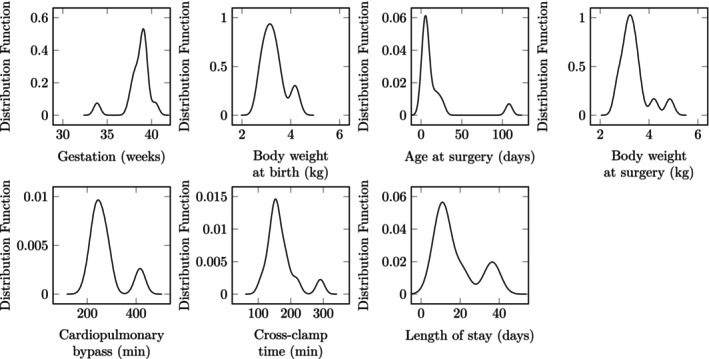
Distributions of patient demographics and surgical outcomes.

Fourteen patients met inclusion criteria. Demographics included mother's gestation age: 38.5 [range: 33.9–40.4] weeks, sex: 5/14 (in line with the known higher prevalence in males^40^), birth weight: 3.29 ± 0.46 kg, age at surgery: 7 [range: 2–108] days, weight at surgery: 3.37 ± 0.57 kg. Outcomes include cardiopulmonary bypass time: 255.5 [range: 199–423] min, cross‐clamp time: 156 [range: 111–291] min, and postoperative length of stay: 12.5 [range: 7–38] days. Distributions of continuous demographics and outcomes are presented in Figure 2. All patients were successfully discharged.

### 2.2 Data preprocessing

There were no measurements for 10.97% of the time‐period under investigation before the first measurement or after the last true measurement. This represents the discrepancies associated with later transfer of patients from the operating room to the PCICU, and earlier discharge. For the remaining time between the first and last recorded measurements for a given signal, 0.63% and 2.63% of the data were identified as dropouts and outliers, respectively. Thus, the monitoring platform and data capture system are extremely reliable, and the data integrity is high.

### 2.3 Trajectory maps

**FIGURE 3:**
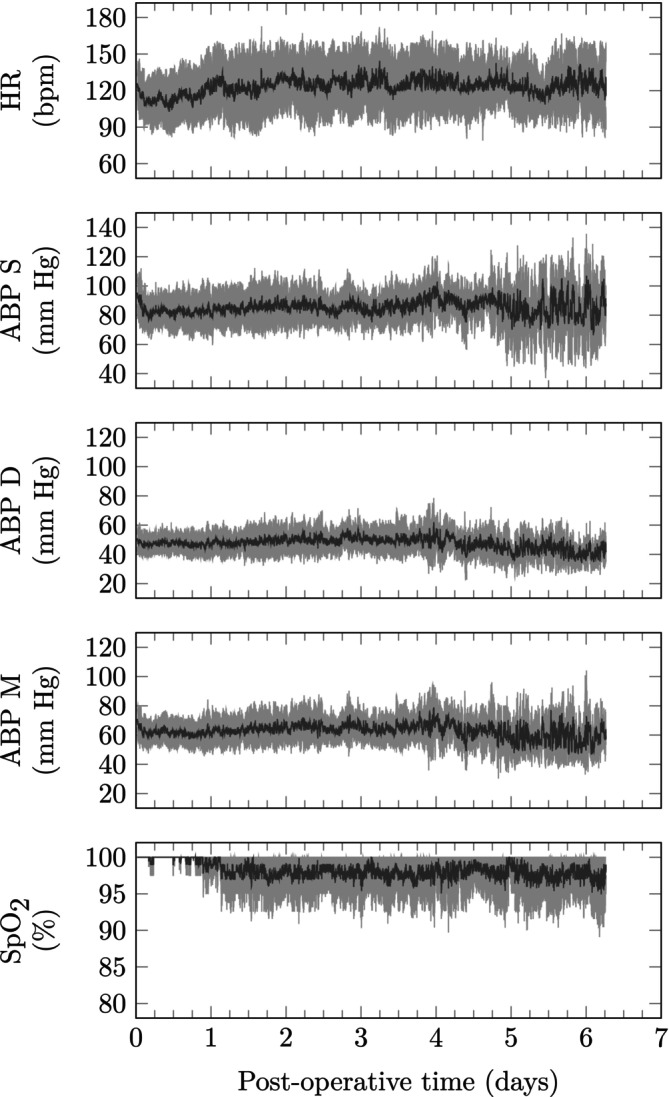
Trajectory map for all arterial switch operation patients. ABP, arterial blood pressure; pulse oximetry, pulse oximetry.

Trajectory maps for the hemodynamic variables using data from all patients are presented in Figure 3. Overall, the heart rate appears relatively static whereas the arterial blood pressure (ABP) exhibits a drop and recovery over the first day. Cardiopulmonary bypass is known to induce low cardiac output after congenital heart surgery and these dynamics are consistent with previous reports of postoperative monitoring of ASO patients.^41^ The drop in SpO_2_ over the 7‐day period is attributed to the expected increased oxygen demand as the patient recovers and is weaned off vasoactive and ventilator support.

### 2.4 Normalization to the baseline trajectory

**FIGURE 4:**
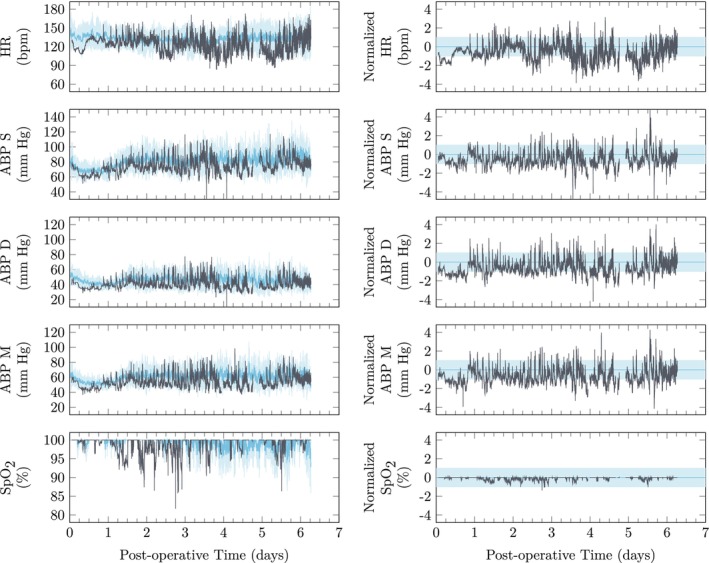
A representative example of normalizing the baseline trajectory and associated variability via batch‐wise unfolding. ABP, arterial blood pressure; pulse oximetry, pulse oximetry.

Postoperative monitoring processes are inherently dynamic as patients recover from surgical intervention. Batch‐wise unfolding is used to correct the baseline trajectory and associated variation. Using leave‐one‐out cross‐validation, an example of this normalization process is presented in Figure 4. In particular, this normalization process removes the low cardiac output in the first postoperative day that is part of the hemodynamics expected for this recovery process.

### 2.5 Comparison of LV techniques

**FIGURE 5:**
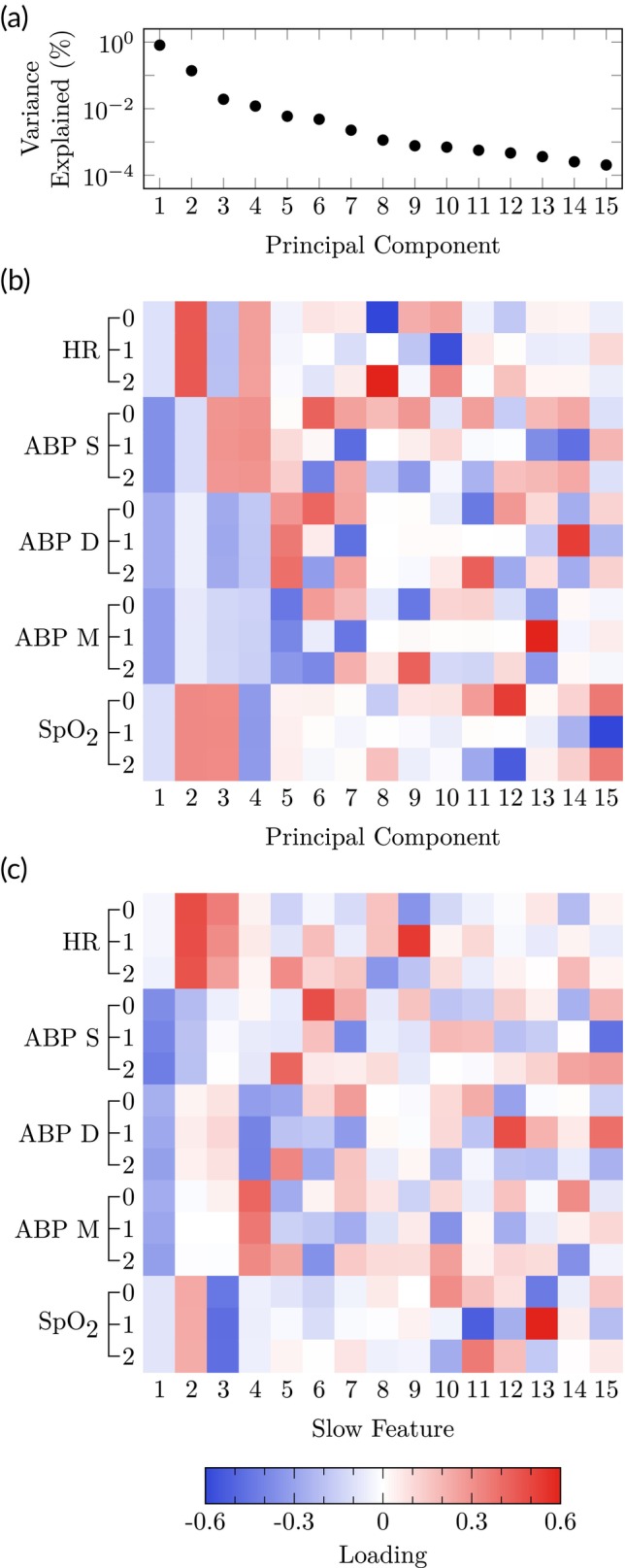
Introspection into latent variable (LV) models identified for postoperative monitoring: (a) Percent variance explained for each LV in the principal component analysis (PCA) models, (b) the loadings for all original variables in each principal component for the PCA models, (c) the loadings for all original variables in each slow feature for the SFA models. In (B,C), loadings are normalized such that the loading vector for each LV has unit magnitude. ABP, arterial blood pressure; pulse oximetry, pulse oximetry.

**FIGURE 6:**
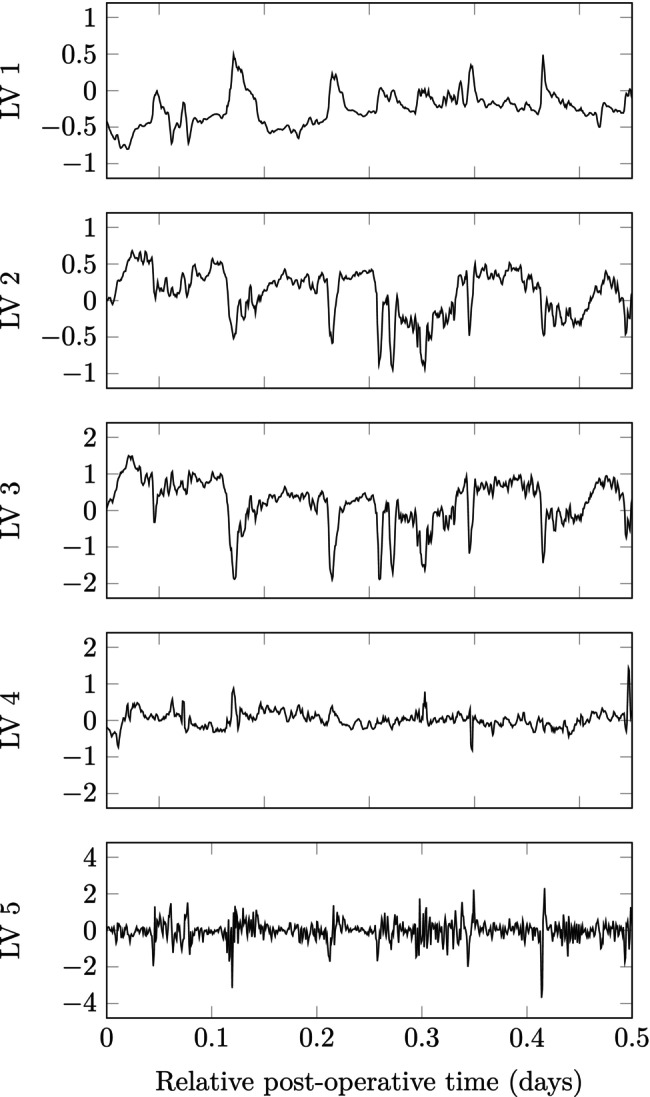
Individual latent variable from slow feature analysis for Patient 1 over a truncated time window.

PCA and SFA models were identified in Figure 5 using data from all patients and augmenting the data matrix with two time‐lagged samples for each hemodynamic variable. The PCA model does not extract dynamic information until LV 5 as seen from equal weighting of time lags within each hemodynamic variable. In contrast, SFA extracts dynamic information starting in LV 1. The ability of SFA to rank LVs by “slowness” is further demonstrated in Figure 6 where higher LV numbers correspond to higher frequencies.

### 2.6 Statistical process monitoring

**FIGURE 7:**
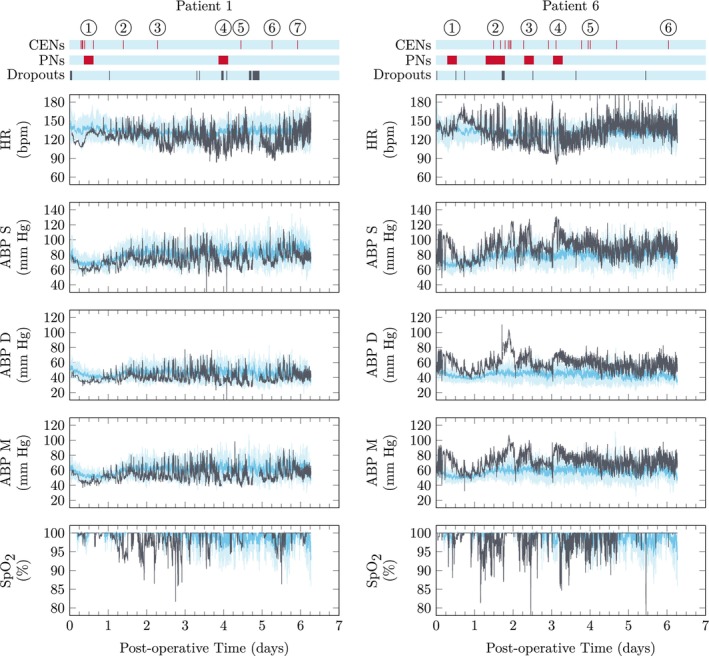
Postoperative monitoring for two patients. In each panel, the darker blue line indicates the mean trajectory of the patients other than the current patient and the lighter blue‐shaded region indicates the region within one standard deviation of this group. The dark gray lines indicate the measurements for the current patient. Spark charts for the clinical notes and dropouts are also provided. Patient 1 adheres to the trajectory well whereas Patient 2 deviates more significantly from the expected trajectory. ABP, arterial blood pressure; CEN, clinical event note; HR, heart rate; PN, progress note; SpO_2_, pulse oximetry.

**FIGURE 8:**
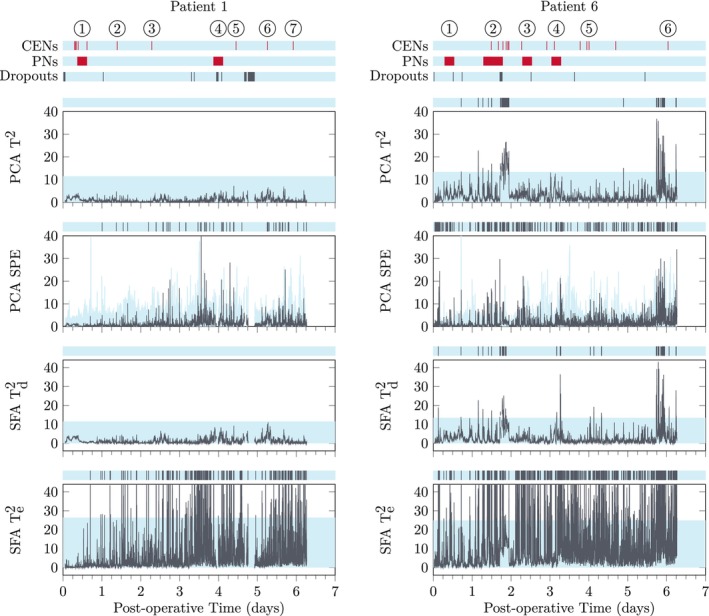
Control charts based on PCA or SFA for the patients are presented in Figure 7. The light‐blue‐shaded regions represent the 99th quantile for each statistic. The spark chart above each subplot represents regions that have crossed the threshold in the corresponding subplot. ABP, arterial blood pressure; CEN, clinical event note; HR, heart rate; PCA, principal component analysis; PN, progress note; SFA, slow feature analysis; SpO_2_, pulse oximetry.

Using leave‐one‐out cross validation as described in Section 5, examples of patients that adhere well and do not adhere well to the trajectories of their peers are provided in Figures 7 and 8.

Patient 1 is well‐controlled and only has fleeting deviations above the PCA $T^{2}$ and SFA $T^{2,d}$ charts. The PN for event 1 describes the patient as remaining “critically ill overnight”, which may simply reflect the normal recovery dynamics. The PN for event 4 describes a narrowing in the superior vena cava that was corrected surgically on the following day. Events 2, 3, 5, and 6 indicate brief changes in the heart rate or blood pressures that do not significantly affect the overall trajectory. Event 7 indicates intermittent premature ventricular contractions that likely require additional features in the SPM framework for elucidation.

Patient 6 does not adhere to the trajectory as well. Events 1–4 indicate extensive agitation and elevated blood pressures even when calm, requiring more frequent analgesia and sedation. CENs at event 5 indicate brief periods of agitation or univariate signals not staying within physician‐directed ranges. Event 6 indicates ST segment changes that may require additional features in the SPM framework for detection. However, there are sustained elevations in the PCA $T^{2}$ and SFA $T^{2,d}$ charts preceding the noted ST segment changes due to elevated diastolic arterial pressures. Moreover, it appears the SFA $T^{2,d}$ is picking up anomalies related to event 4 that are missed by the PCA $T^{2}$ chart.

## 3 DISCUSSION

Trajectory maps can effectively summarize dynamic postoperative recovery processes. These maps can facilitate clinical decision‐making by providing dynamic thresholds and a visual tool that readily displays their current patient on a reference background. SPM methods and multivariate control charts codify the qualitative decision‐making by calculating the statistical adherence to the expected trajectory after discarding noisy directions in the multivariate space. This facilitates the generation of a limited set of control metrics and multivariate alarms that do not rely on clinical staff constantly interpreting individual metrics to try and build “mental” trajectory maps for each patient under their care.

The threshold crossings and statistic changes of the PCA $T^{2}$ and SFA $T^{2,d}$ charts are more qualitatively similar to the clinical notes than the PCA SPE and SFA $T^{2,e}$ chart. This is expected since the major variation (PCA $T^{2}$) and slow duration changes (SFA $T^{2,d}$) should better reflect clinical notetaking practices that avoid recording every fleeting sensor deviation in favor of longer‐term behaviors. The PCA SPE and SFA $T^{2,e}$ charts may become more useful with enhanced filtering prior to construction of the control charts and more patients in the cohort for comparison.

Retrospective analyses and comparison with clinical notes are limited in their ability to assess the impact of such a monitoring statistic or alarm system. Clinical documentation in the PCICU by the physician is only required once per day for each patient. This “daily progress note” is often entered into the medical record hours or days removed from the time the care is performed, with varying levels of details and specificity depending on the clinician.^42^ Many physicians use clinical documentation more to justify medical billing and reimbursement rather than being a detailed reflection of care provided each day.^42^ Additionally, it is likely that significant “clinical events” were not captured by documentation unless a specific procedure is performed that justifies documentation (e.g., patient consent, billing codes for reimbursement). Future work will implement SPM in real‐time and provide real‐time feedback to collect more accurate information regarding the patient's status during deviations from the expected trajectory.

While this approach shows promise for providing PCICU clinical teams with advanced warning systems based on previous patient trajectories, this approach is not without limitations. This approach assumes that all patients recover in the same way from a particular surgery. This assumption may be violated in larger cohorts and subgroups of patient dynamics may emerge. This limitation can be addressed by first clustering patients into multiple groups and developing multiple LV models for each group. Such a multiple model approach is the subject of future work. Furthermore, some *T* ^2^ or *T* ^2,d^ deviations may indicate a faster recovery or different, but acceptable, recovery trajectory. Fault reconstruction or contribution charts^43^ can be used to identify likely root causes of the control chart deviations and certain types of contributions could be ignored if the recovery direction is acceptable. Alternatively, adaptive strategies could be explored that tailor this approach to the patient over time; however, adaptive strategies would need to ensure that long‐term trajectory deviations are clinically acceptable. Finally, other types of CHDs may require more than one surgery, such as with single ventricle palliation. In this case, multiple phases could be considered in the SPM framework.^27^

## 4 CONCLUSIONS

This article describes the translation of SPM techniques developed for batch processes to clinical vital sign monitoring in postoperative patients. By collating information from many different sensors and devices into a single or reduced set of monitoring statistics, clinicians have fewer measurements to visually inspect. Furthermore, by comparing the current patient with a historical record of successful patients in real‐time, clinicians can understand whether deviations out of the desired static range are consistent with previous patients or a deviation that warrants modifications to the current patient's care plan.

## 5 MATERIALS AND METHODS

### 5.1 Patient cohort

All patients undergoing ASO from January 7, 2020 to July 3, 2022 at The Texas Center for Pediatric and Congenital Heart Disease (Austin, TX) were retrospectively reviewed using real‐time, HFDC software (Sickbay™, Medical Informatics, Houston, TX) from postoperative admission to the PCICU and continued for up to 7 days. Continuous monitoring of heart rate (bpm), invasive APBs (systolic, diastolic, and mean; mmHg), and pulse oximetry saturations (SpO_2_, %) obtained from Phillips© telemetry monitors (Koninklijke Philips N.V., Amsterdam, Netherlands) and stored by Sickbay™ (Medical Informatics, Houton, TX) were used for model development. The only exclusion criterion was insufficient data capture, assessed by dropping out of any sensor for more than 10% of the total monitoring time, which did not affect any patient in this cohort. All information regarding human participants were obtained in accordance with the ethical standards of the institutional review boards at the participating institutions and with the 1964 Declaration of Helsinki and its later amendments and comparable ethical standards.

### 5.2 Demographic and outcome statistics

Statistics for demographics and outcomes are provided as mean ± standard deviation or median [range].

### 5.3 Encoding clinical events

Retrospective chart review of the electronic medical record was undertaken to obtain deidentified clinical documentation in the form of daily “progress notes” (PNs) and clinical event notes (CENs) for the first 7 postoperative days. PNs are a summary of clinical care documented by the clinician once per day, while CENs capture significant acute changes or interventions documented by the bedside nurse. These were digitized and either assigned a timestamp (when available) or a general time frame (morning, afternoon, evening, night) when clinically relevant observations were documented. These documented observations in PNs and CENs were reviewed for clinical significance/relevance to the hemodynamic trajectories of the patients by the clinical physicians in our research team in a blinded fashion.

### 5.4 Software

All methods and algorithms were implemented in the Julia programming language.

### 5.5 Cross validation

Leave‐one‐out cross validation was employed throughout this investigation, where data from the patient under consideration are not used to construct the control charts used for their assessment. This allows for an independent assessment of each patient and improves generalizability of the results.

### 5.6 Dropout and outlier detection

Postoperative data streams were normalized to the operation end time and sampled at 1 min resolution with linear interpolation. Dropout periods were identified as those time periods with 3 consecutive minutes without a measurement and these dropouts were removed from analysis. Outliers in the historical trajectories were identified and replaced with a windowed median via a Hampel identifier^44^ over centered 11 min windows with an allowed misidentification rate of α=0.05.

### 5.7 Multiway unfolding and normalization

As in batch process monitoring, patient monitoring can be characterized by a three‐dimensional array of size I×J×K, where I is the number of patients, J is the number of signals, and K is the number of sampling times. It is important to note that the number of signals incorporates any time‐lagged signals. We first perform a batch‐wise unfolding to normalize the data to zero mean and unit variance along each variable at every sampling time,^28^ except for the SpO_2_ readings that were normalized to a value of 100% and standard deviation of 5% for each time point. The data is then unfolded variable‐wise and standard LV techniques are applied to this IK×J matrix denoted as X.

### 5.8 Principal component analysis

Let, $B=X^{T}X$ be the covariance matrix of the unfolded data matrix. Singular value decomposition (SVD) on B results in the decomposition $B=U\Lambda^{2}U^{T}$ and the score matrix $S^{\mathrm{PCA}}$ is given by $S^{\mathrm{PCA}}=\mathrm{XU}\Lambda^{-1}$. The columns of $S^{\mathrm{PCA}}$ are the scores of the principal components organized by the amount of variance they explain and only the first c principal components are retained in $S_{1:c}^{\mathrm{PCA}}$ for construction of the PCA control charts. Herein, c=3 was chosen by the L‐curve method and resulted in explaining >98% of the variance for each cross‐validation cohort.

### 5.9 Slow feature analysis

Following the first round of SVD described for PCA, the data in X are then sphered $Z=\mathrm{XU}\Lambda^{-1}$ and a new covariance matrix $A={\dot{Z}}^{T}\dot{Z}$ is constructed from the first time‐derivatives of the sphered signals in Z. Here, the first derivatives are approximated with backward finite differences. Then, A is decomposed with SVD, yielding $A=P^{T}\Omega P$ and the slow feature scores are given by $S^{\mathrm{SFA}}=\mathrm{XU}\Lambda^{-1}P$. The columns of $S^{\mathrm{SFA}}$ are the slow features organized by increasing slowness and a partition $S^{\mathrm{SFA}}=\left[S_{1:d}^{\mathrm{SFA}}\;S_{d+1:j}^{\mathrm{SFA}}\right]$ is created to separate features into those associated with fast anomalies versus slow operating point changes. Herein, d was chosen equal to the number of components c used in the corresponding PCA chart.

### 5.10 Control charts

PCA monitoring employs both Hoetelling $T^{2}$ (or simply $T^{2}$) charts and squared prediction error (SPE) charts for detecting deviations in patient adherence to the typical trajectory. $T^{2}$ are constructed to monitor deviations from the average trajectory. For PCA models, the $T^{2}$ statistic at time k is calculated as

$$T^{2}\left(k\right)={\tilde{s}}_{1:c}{\left(k\right)}^{T}{\left[{\left(S_{1:c}^{\mathrm{PCA}}\right)}^{T}S_{1:c}^{\mathrm{PCA}}\right]}^{-1}{\tilde{s}}_{1:c}\left(k\right)\sim \chi_{c}^{2}$$

where the ~ accent represents the score from the current patient under investigation.^30^, ^43^, ^45^ SPE charts are constructed to monitor the variability that violates the normal process correlation for PCA models. The SPE statistic at time k is calculated as

$$\mathrm{SPE}\left(k\right)={\tilde{s}}_{c+1:j}{\left(k\right)}^{T}{\tilde{s}}_{c+1:j}\left(k\right)\sim \frac{v_{k}}{2m_{k}}\chi_{2m_{k}^{2}/v_{k}}^{2}$$

where $m_{k}$ and $v_{k}$ are the mean and variance, respectively, of the SPE at time k in the training set.^30^ Here, we employ a windowing procedure over a centered 5 min window as suggested previously^30^ to smooth the SPE limits for small sample sizes.

SFA monitoring employs $T^{2}$ charts for both the slow ($T^{2,d}$) and fast ($T^{2,e}$) LVs. The $T^{2,d}$ and $T^{2,e}$ statistics at time k are calculated as

$$\begin{array}{c} T_{d}^{2,d}\left(k\right)={\tilde{s}}_{1:d}{\left(k\right)}^{T}{\tilde{s}}_{1:d}\left(k\right)\sim \chi_{d}^{2}\\ T^{2,e}\left(k\right)={\tilde{s}}_{d+1:J}{\left(k\right)}^{T}{\tilde{s}}_{d+1:J}\left(k\right)\sim \chi_{J-d}^{2} \end{array}$$

where the property $E\left[s^{T}s\right]=I$ is utilized.

Thresholds for all control charts are presented for α=0.01.

## AUTHOR CONTRIBUTIONS

**Daniel P. Howsmon:** Conceptualization; data curation; formal analysis; funding acquisition; investigation; methodology; project administration; software; visualization; writing—original draft; writing—review and editing. **Matthew F. Mikulski:** Conceptualization; data curation; investigation; project administration; writing—review and editing. **Nikhil Kabra:** Formal analysis; investigation; visualization; writing—review and editing. **Joyce Northrup:** Investigation; resources; supervision; validation; writing—review and editing. **Daniel Stromberg:** Investigation; resources; supervision; validation; writing—review and editing. **Charles D. Fraser:** Investigation; resources; supervision; validation; writing—review and editing. **Carlos M. Mery:** Investigation; resources; supervision; validation; writing—review and editing. **Richard P. Lion:** Conceptualization; data curation; investigation; project administration; resources; supervision; validation; writing—review and editing.

## FUNDING INFORMATION

DPH is supported by startup funds from the Tulane University.

## CONFLICT OF INTEREST STATEMENT

The authors declare no conflicts of interest.

### PEER REVIEW

The peer review history for this article is available at https://www.webofscience.com/api/gateway/wos/peer‐review/10.1002/btm2.10679.

## PATIENT CONSENT STATEMENT

Due to the retrospective and observational nature of the study, patient consent was not required by the University of Texas Institutional Review Board (Study no. 00001279, approved on November 11, 2021). All patient information was de‐identified and stored on a password‐protected computer per institutional IRB protocols/standards.

## Supporting information


**Data S1.** Supporting Information.

## Data Availability

The raw data are not publicly available due to patient privacy restrictions.
