# Supplementary material for: Statistical process monitoring creates a hemodynamic trajectory map after pediatric cardiac surgery: A case study of the arterial switch operation
Source: Bioeng Transl Med. 2024 May 15;9(6):e10679. doi: 10.1002/btm2.10679 (PMC11558195; doi:10.1002/btm2.10679)
Supplement: Supplementary file 1 — Data S1. Supporting Information. [file BTM2-9-e10679-s001.pdf]

Supplementary Material
Statistical Process Monitoring Creates a Hemodynamic Trajectory Map after Pediatric Cardiac Surgery: A Case Study of the Arterial Switch Operation

Daniel P. Howsmon PhD[1,*], Matthew F. Mikulski MD MSHCT[2,3,4], Nikhil Kabra[5], Joyce Northrup RN[2], Daniel Stromberg MD[2,3,4], Charles D. Fraser, Jr MD[2,3,4], Carlos M. Mery MD MPH[2,3,4], and Richard P. Lion DO MPH[2,4,*,**]

[1]Department of Chemical and Biomolecular Engineering, Tulane University, New Orleans, LA, USA
[2]Texas Center for Pediatric and Congenital Heart Disease, University of Texas Health Austin and Dell Children's Medical Center, Austin, TX, USA
[3]Department of Surgery and Perioperative Care, Dell Medical School, The University of Texas at Austin, Austin, TX, USA
[4]Department of Pediatrics, Dell Medical School, The University of Texas at Austin, Austin, TX, USA
[5]Chandra Department of Electrical and Computer Engineering, the University of Texas at Austin, Austin, TX, USA
[*]Corresponding author
[**]Present affiliation: Department of Pediatrics, Division of Critical Care Medicine, Texas Children's Hospital, Baylor College of Medicine, Houston, TX, USA

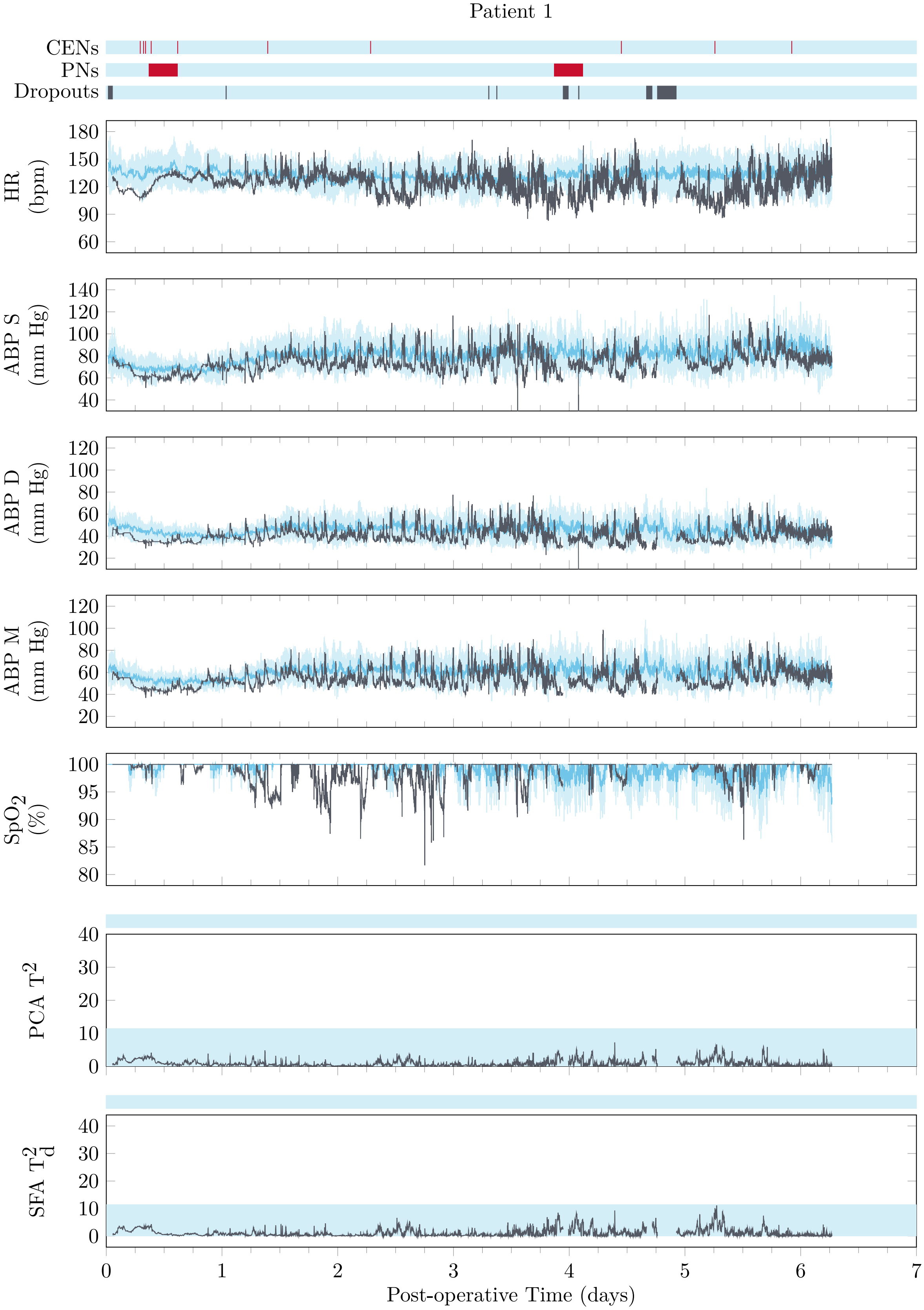

Figure S1: Post-operative monitoring for Patient 1. In each panel, the darker blue line indicates the mean trajectory of the patients other than the current patient and the lighter blue shaded region indicates the region within one standard deviation of this group. The dark grey lines indicate the measurements for the current patient. Spark charts for the clinical event notes, progress notes, and dropouts are also provided.

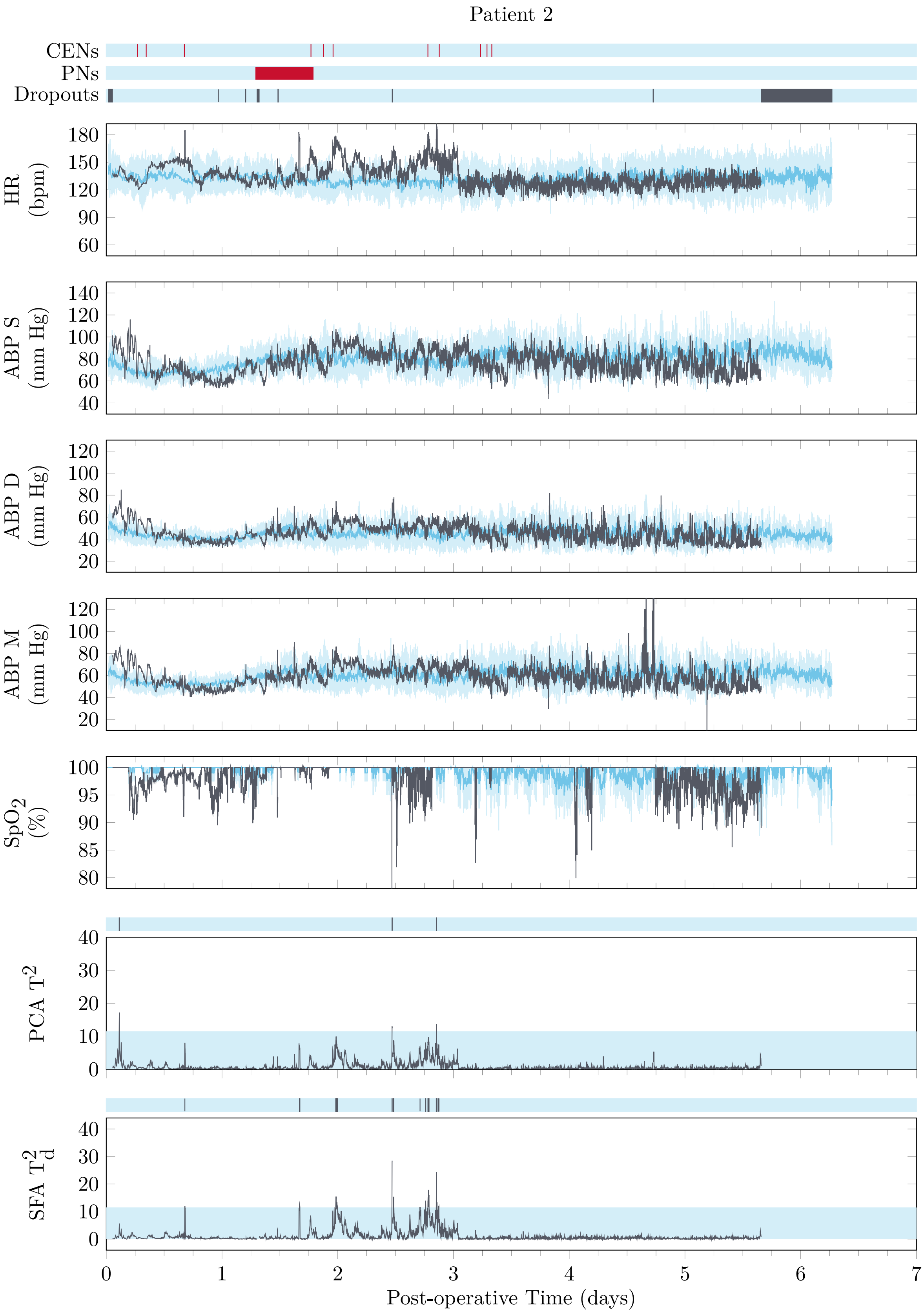

Figure S2: Post-operative monitoring for Patient 2. In each panel, the darker blue line indicates the mean trajectory of the patients other than the current patient and the lighter blue shaded region indicates the region within one standard deviation of this group. The dark grey lines indicate the measurements for the current patient. Spark charts for the clinical event notes, progress notes, and dropouts are also provided.

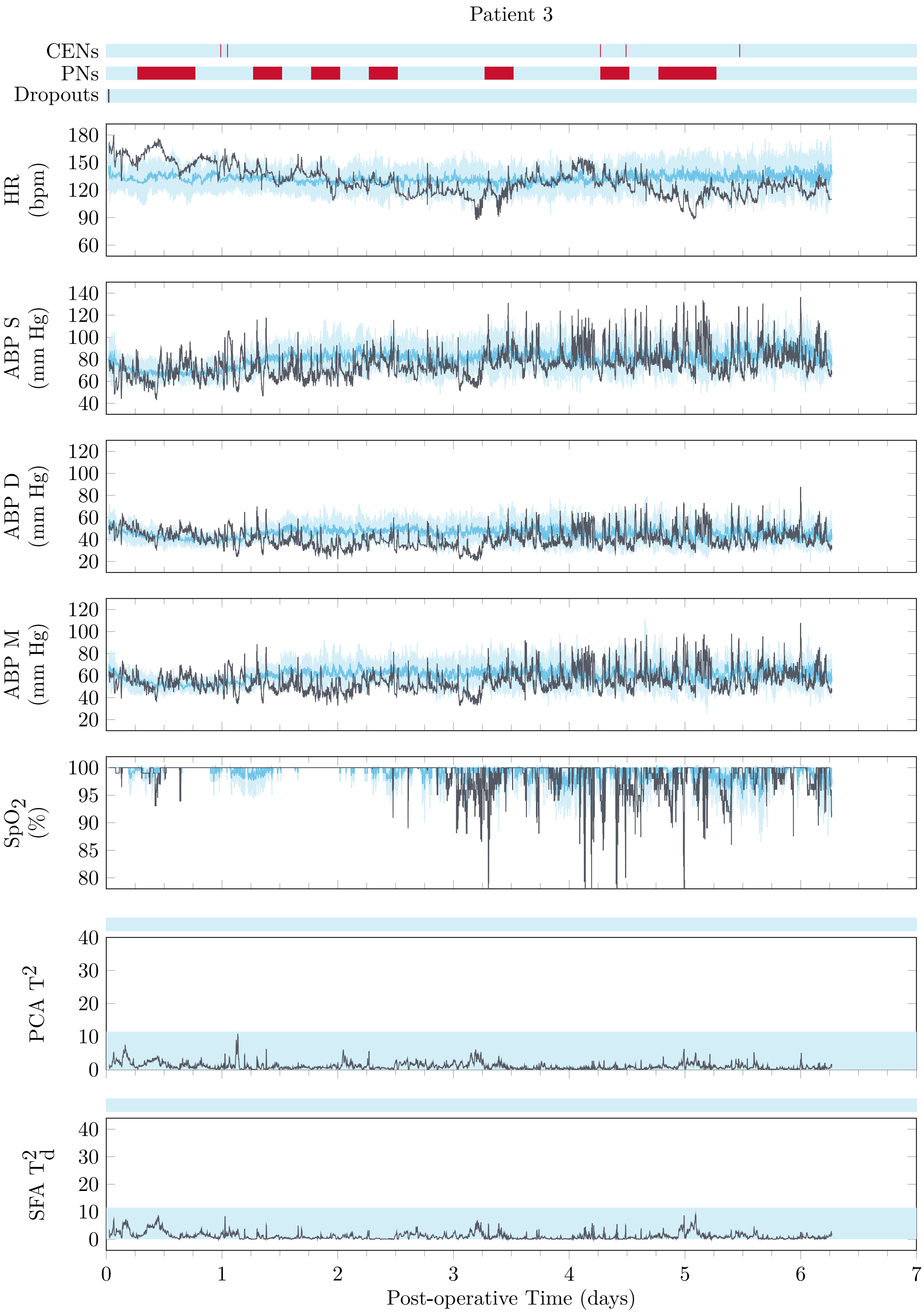

Figure S3: Post-operative monitoring for Patient 3. In each panel, the darker blue line indicates the mean trajectory of the patients other than the current patient and the lighter blue shaded region indicates the region within one standard deviation of this group. The dark grey lines indicate the measurements for the current patient. Spark charts for the clinical event notes, progress notes, and dropouts are also provided.

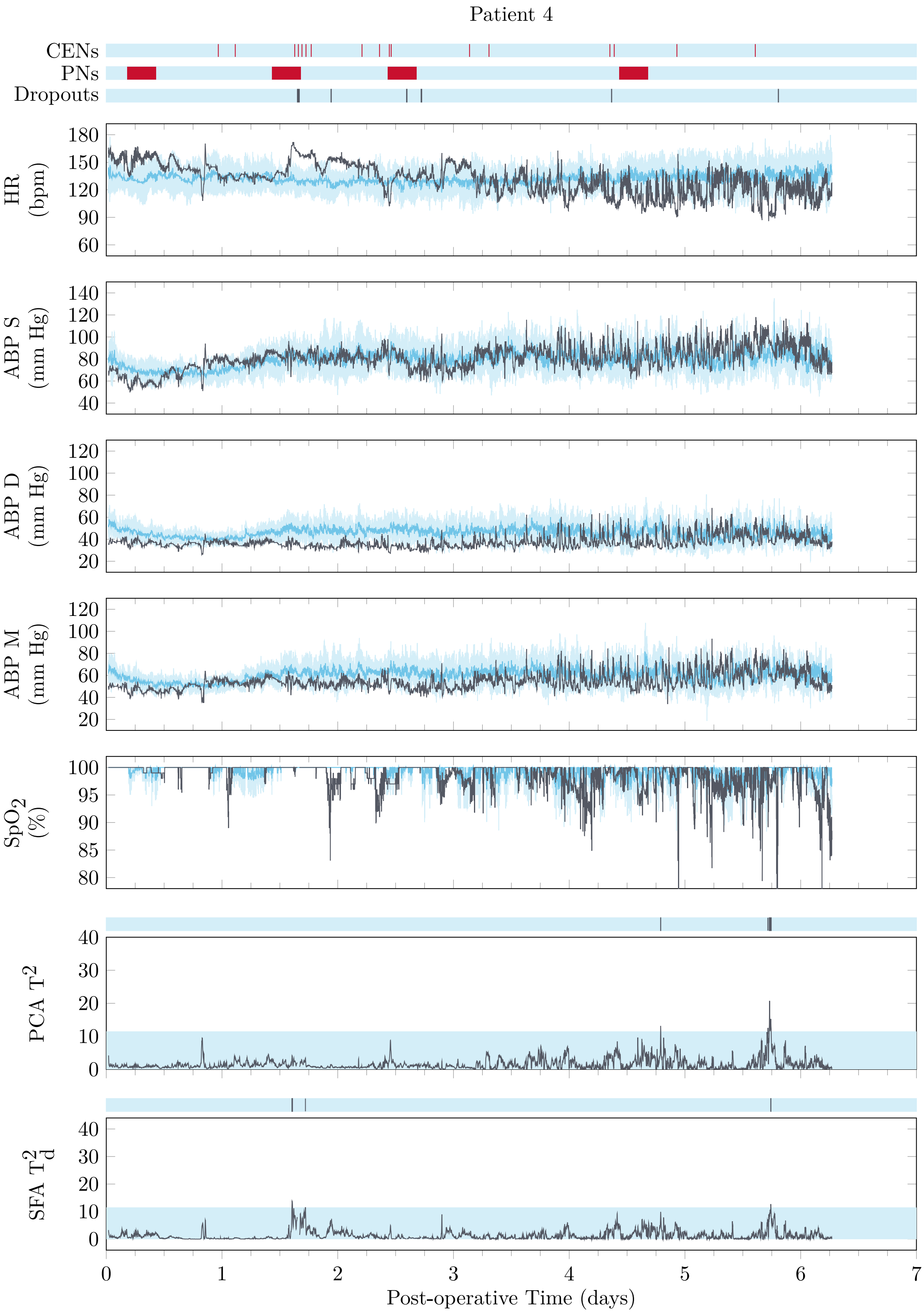

Figure S4: Post-operative monitoring for Patient 4. In each panel, the darker blue line indicates the mean trajectory of the patients other than the current patient and the lighter blue shaded region indicates the region within one standard deviation of this group. The dark grey lines indicate the measurements for the current patient. Spark charts for the clinical event notes, progress notes, and dropouts are also provided.

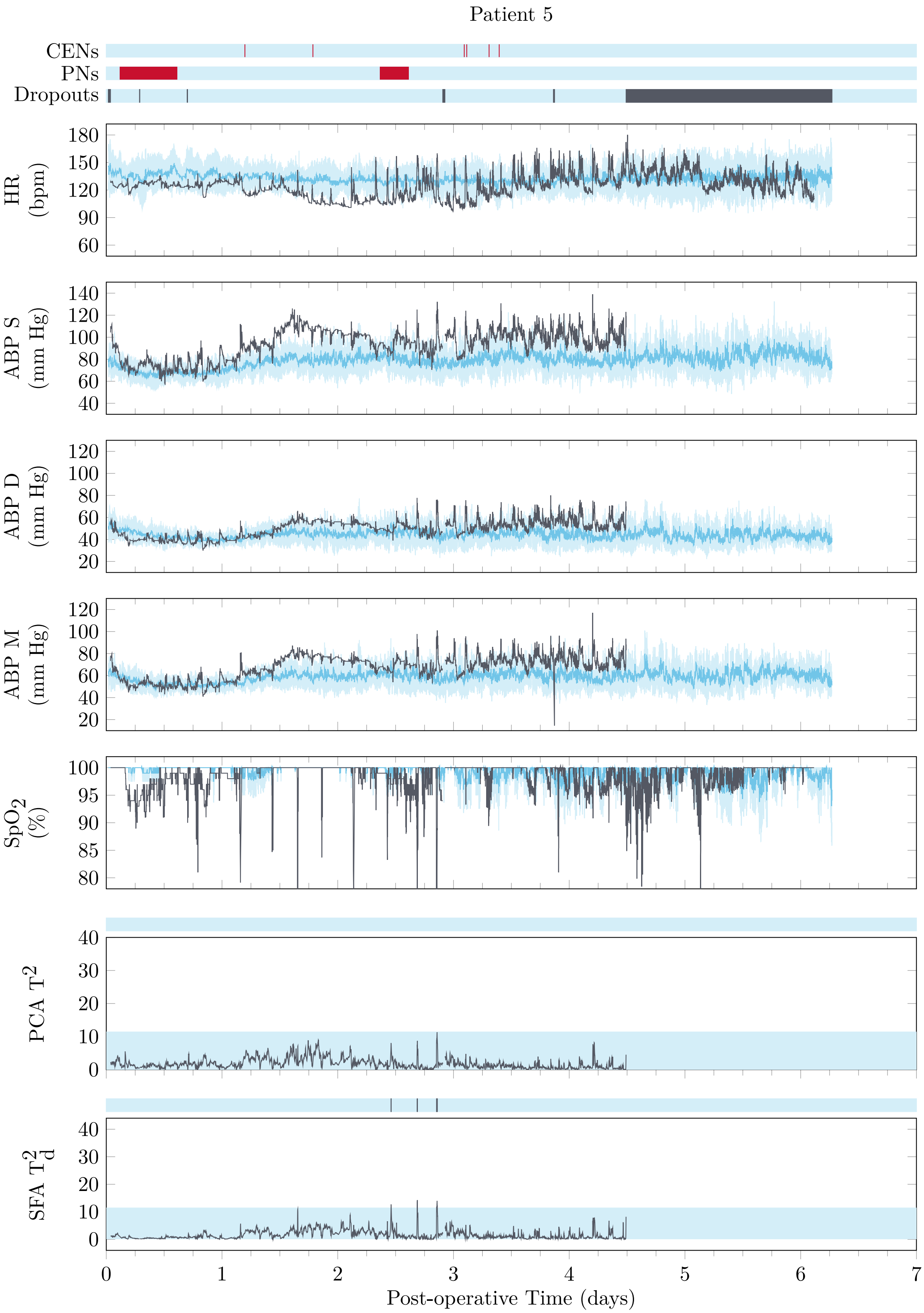

Figure S5: Post-operative monitoring for Patient 5. In each panel, the darker blue line indicates the mean trajectory of the patients other than the current patient and the lighter blue shaded region indicates the region within one standard deviation of this group. The dark grey lines indicate the measurements for the current patient. Spark charts for the clinical event notes, progress notes, and dropouts are also provided.

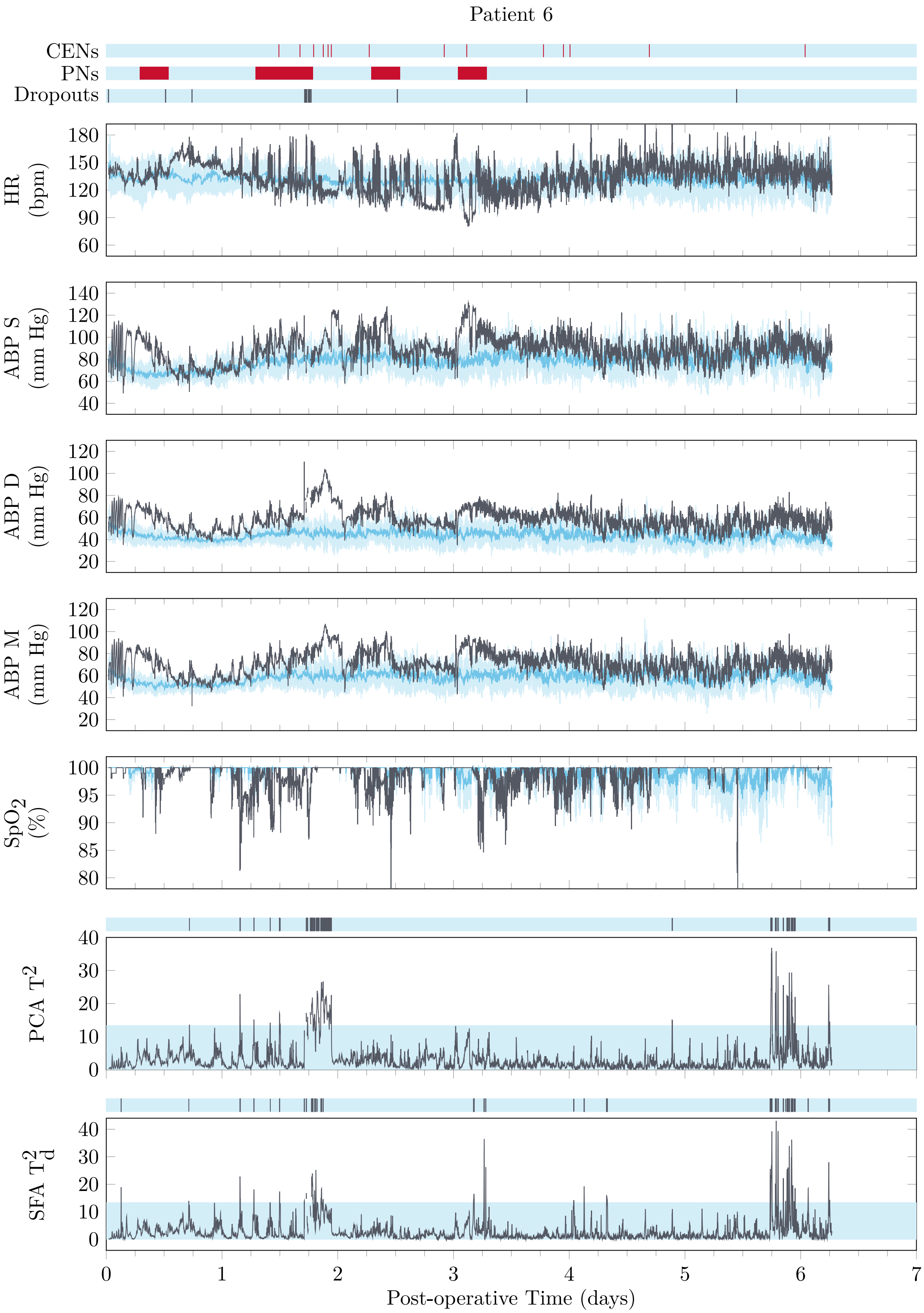

Figure S6: Post-operative monitoring for Patient 6. In each panel, the darker blue line indicates the mean trajectory of the patients other than the current patient and the lighter blue shaded region indicates the region within one standard deviation of this group. The dark grey lines indicate the measurements for the current patient. Spark charts for the clinical event notes, progress notes, and dropouts are also provided.

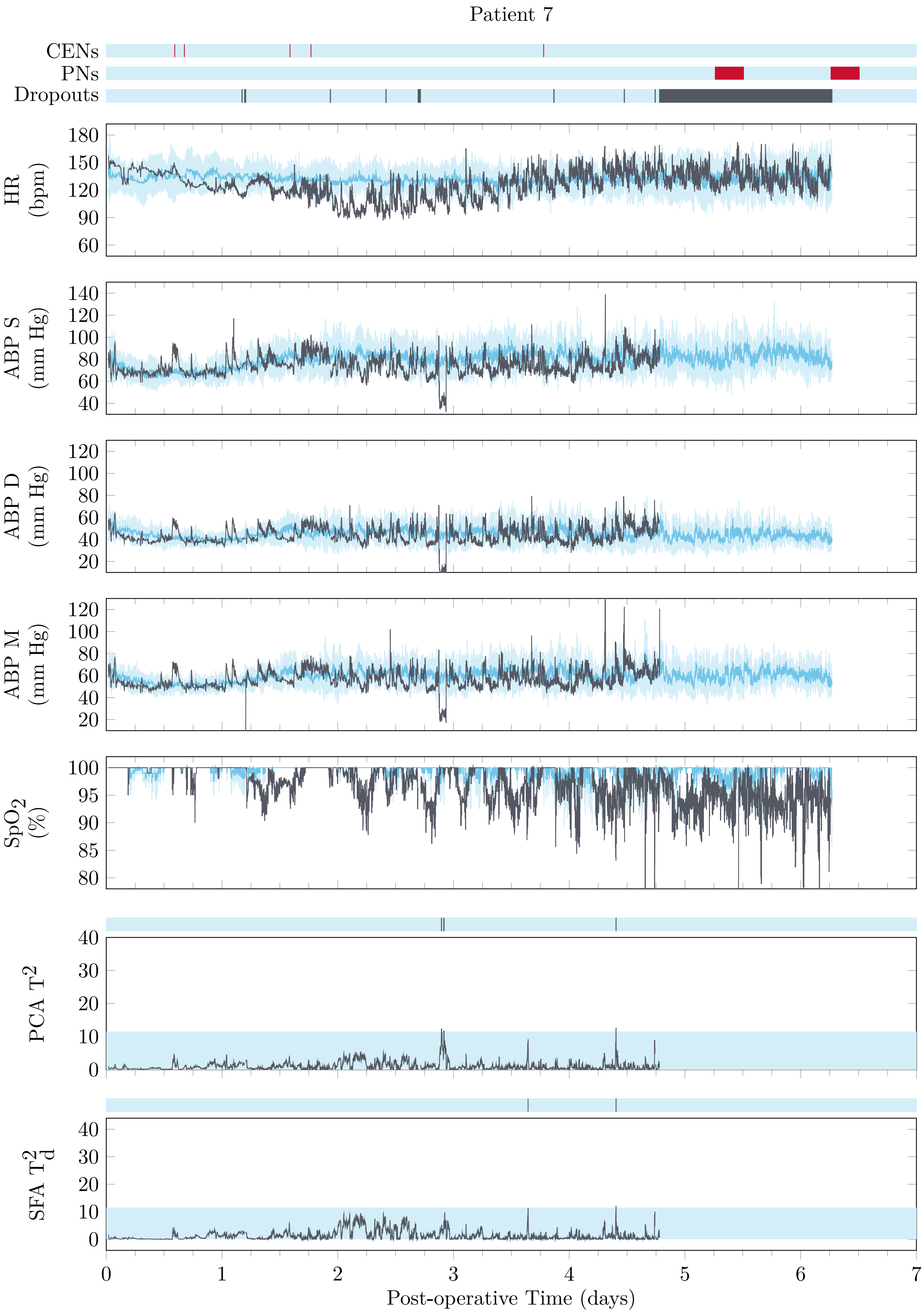

Figure S7: Post-operative monitoring for Patient 7. In each panel, the darker blue line indicates the mean trajectory of the patients other than the current patient and the lighter blue shaded region indicates the region within one standard deviation of this group. The dark grey lines indicate the measurements for the current patient. Spark charts for the clinical event notes, progress notes, and dropouts are also provided.

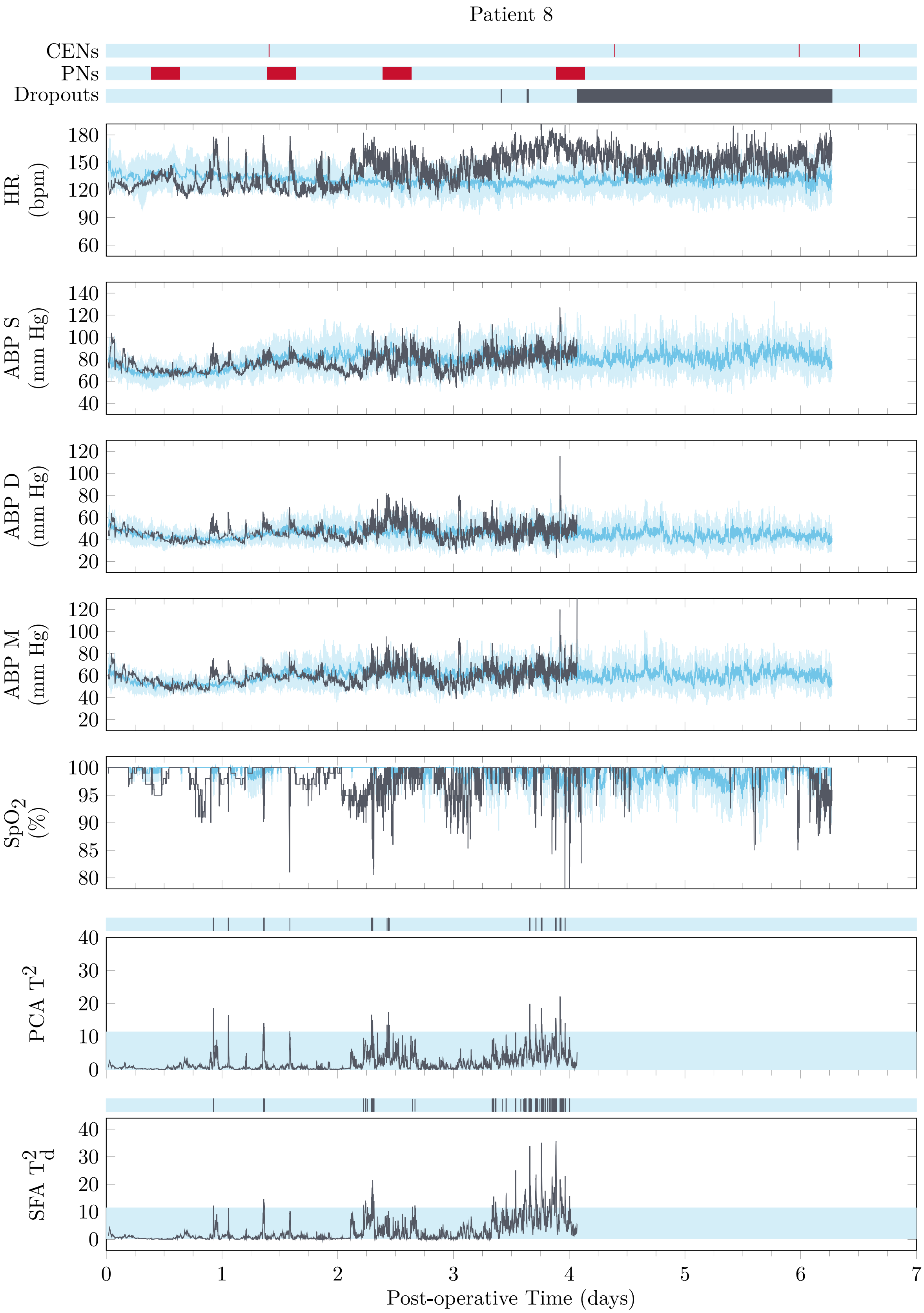

Figure S8: Post-operative monitoring for Patient 8. In each panel, the darker blue line indicates the mean trajectory of the patients other than the current patient and the lighter blue shaded region indicates the region within one standard deviation of this group. The dark grey lines indicate the measurements for the current patient. Spark charts for the clinical event notes, progress notes, and dropouts are also provided.

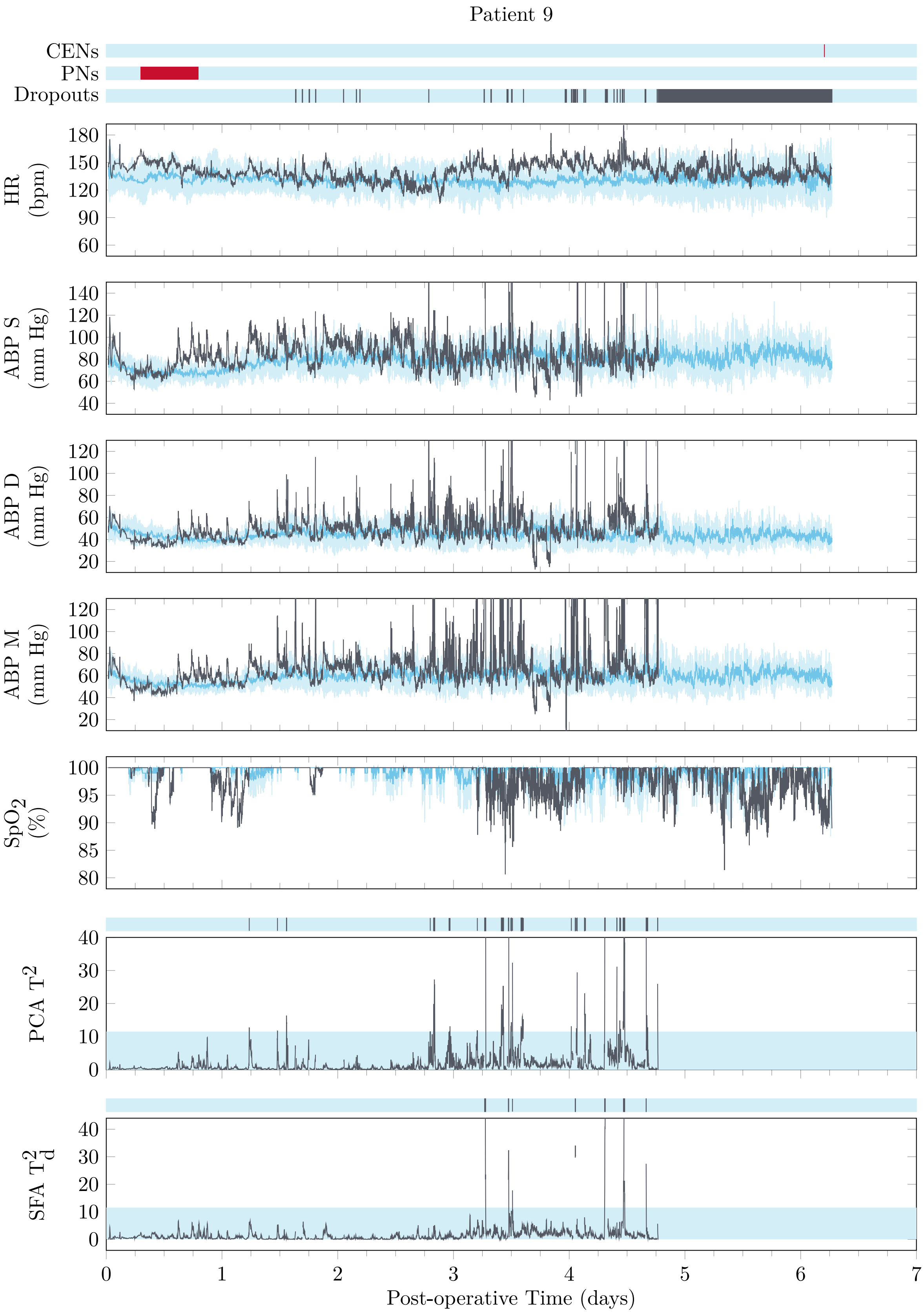

Figure S9: Post-operative monitoring for Patient 9. In each panel, the darker blue line indicates the mean trajectory of the patients other than the current patient and the lighter blue shaded region indicates the region within one standard deviation of this group. The dark grey lines indicate the measurements for the current patient. Spark charts for the clinical event notes, progress notes, and dropouts are also provided.

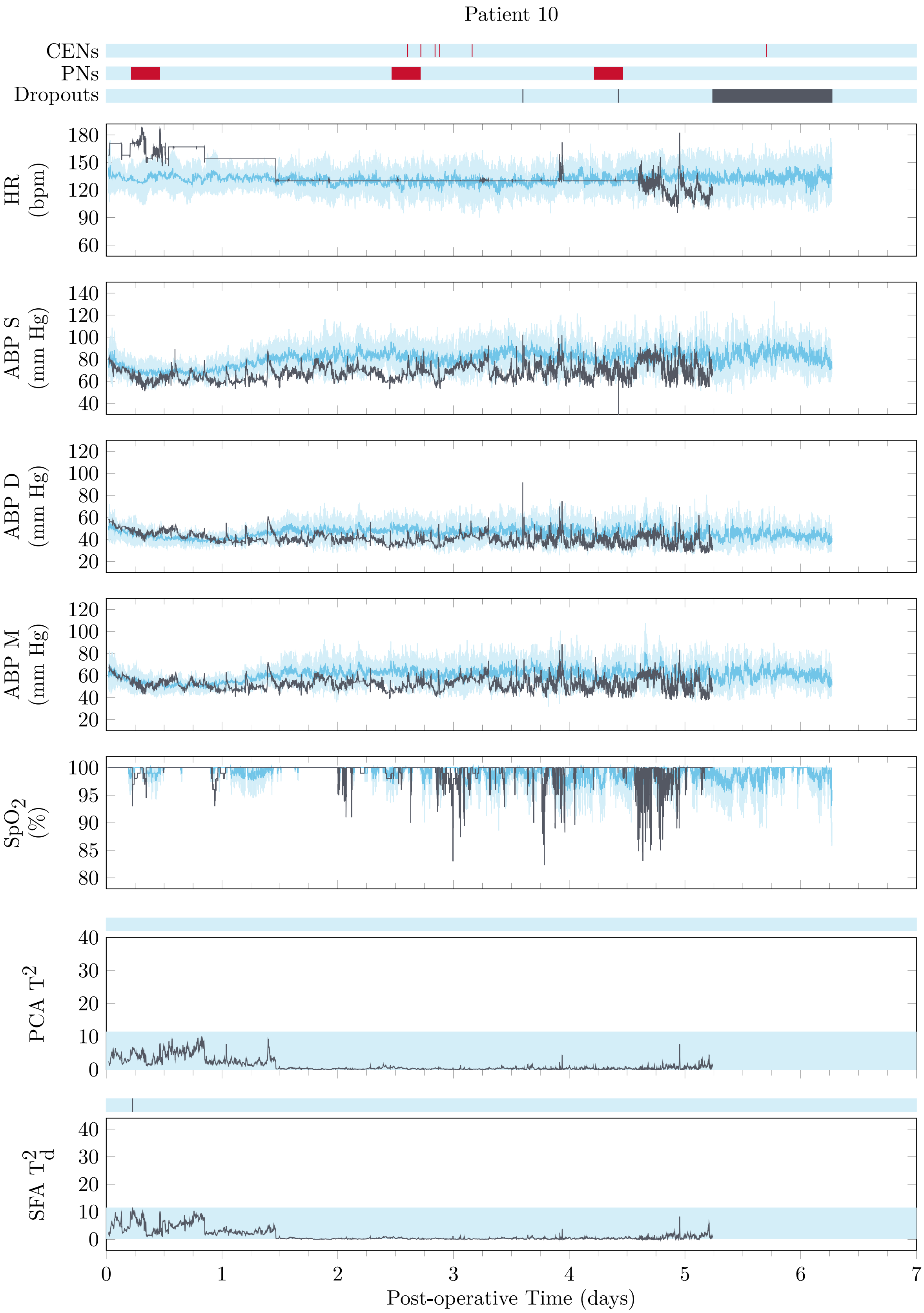

Figure S10: Post-operative monitoring for Patient 10. In each panel, the darker blue line indicates the mean trajectory of the patients other than the current patient and the lighter blue shaded region indicates the region within one standard deviation of this group. The dark grey lines indicate the measurements for the current patient. Spark charts for the clinical event notes, progress notes, and dropouts are also provided.

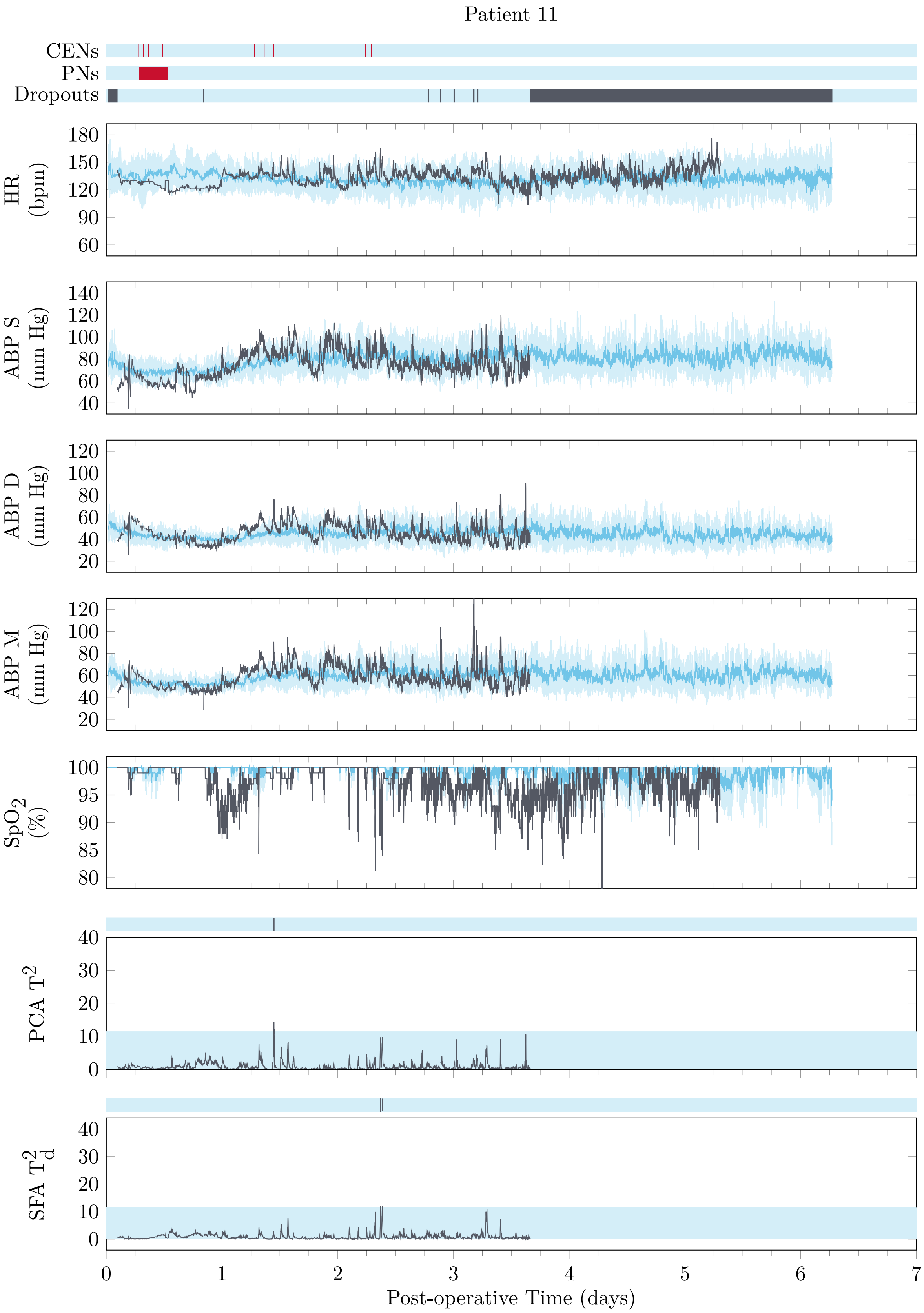

Figure S11: Post-operative monitoring for Patient 11. In each panel, the darker blue line indicates the mean trajectory of the patients other than the current patient and the lighter blue shaded region indicates the region within one standard deviation of this group. The dark grey lines indicate the measurements for the current patient. Spark charts for the clinical event notes, progress notes, and dropouts are also provided.

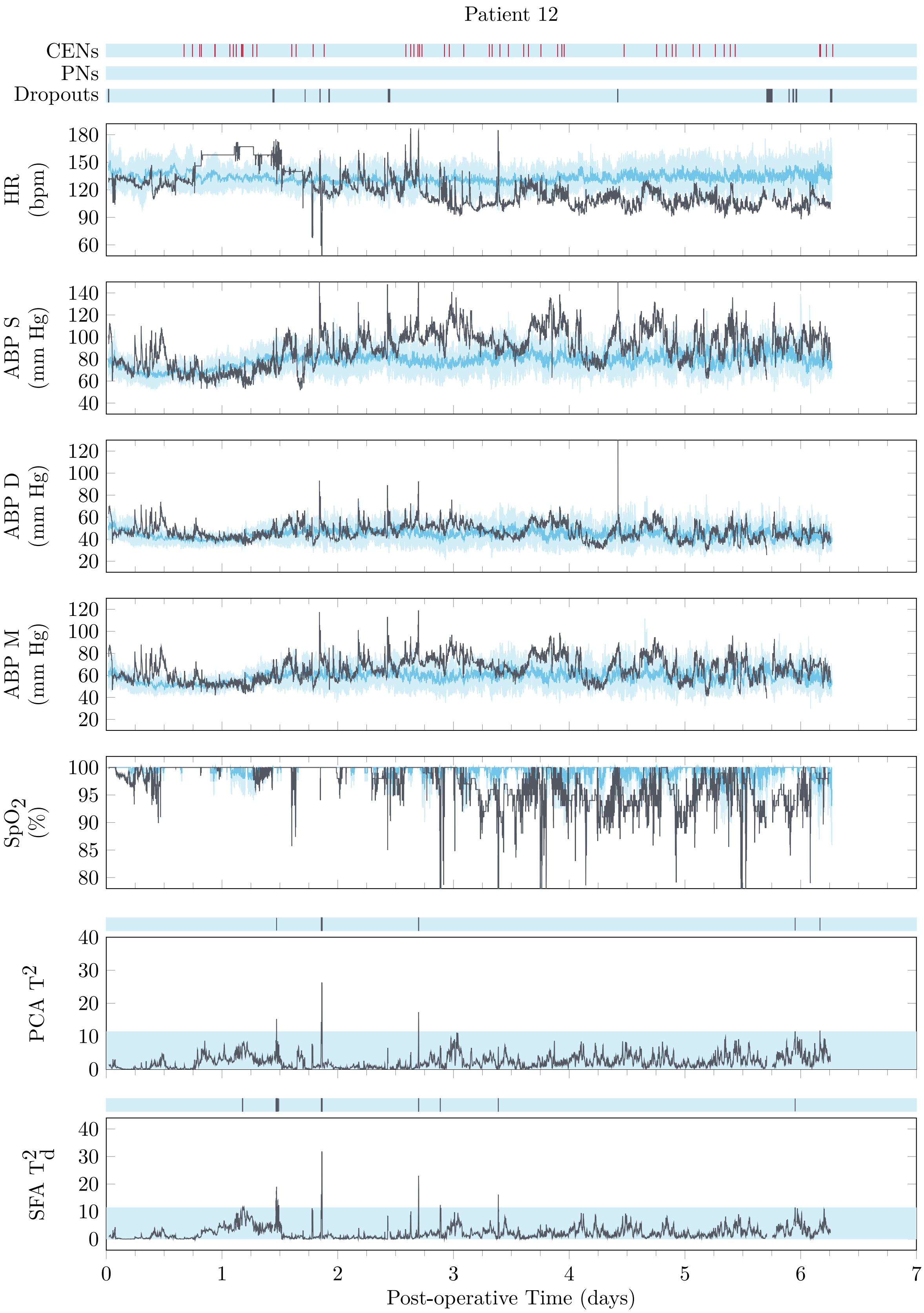

Figure S12: Post-operative monitoring for Patient 12. In each panel, the darker blue line indicates the mean trajectory of the patients other than the current patient and the lighter blue shaded region indicates the region within one standard deviation of this group. The dark grey lines indicate the measurements for the current patient. Spark charts for the clinical event notes, progress notes, and dropouts are also provided.

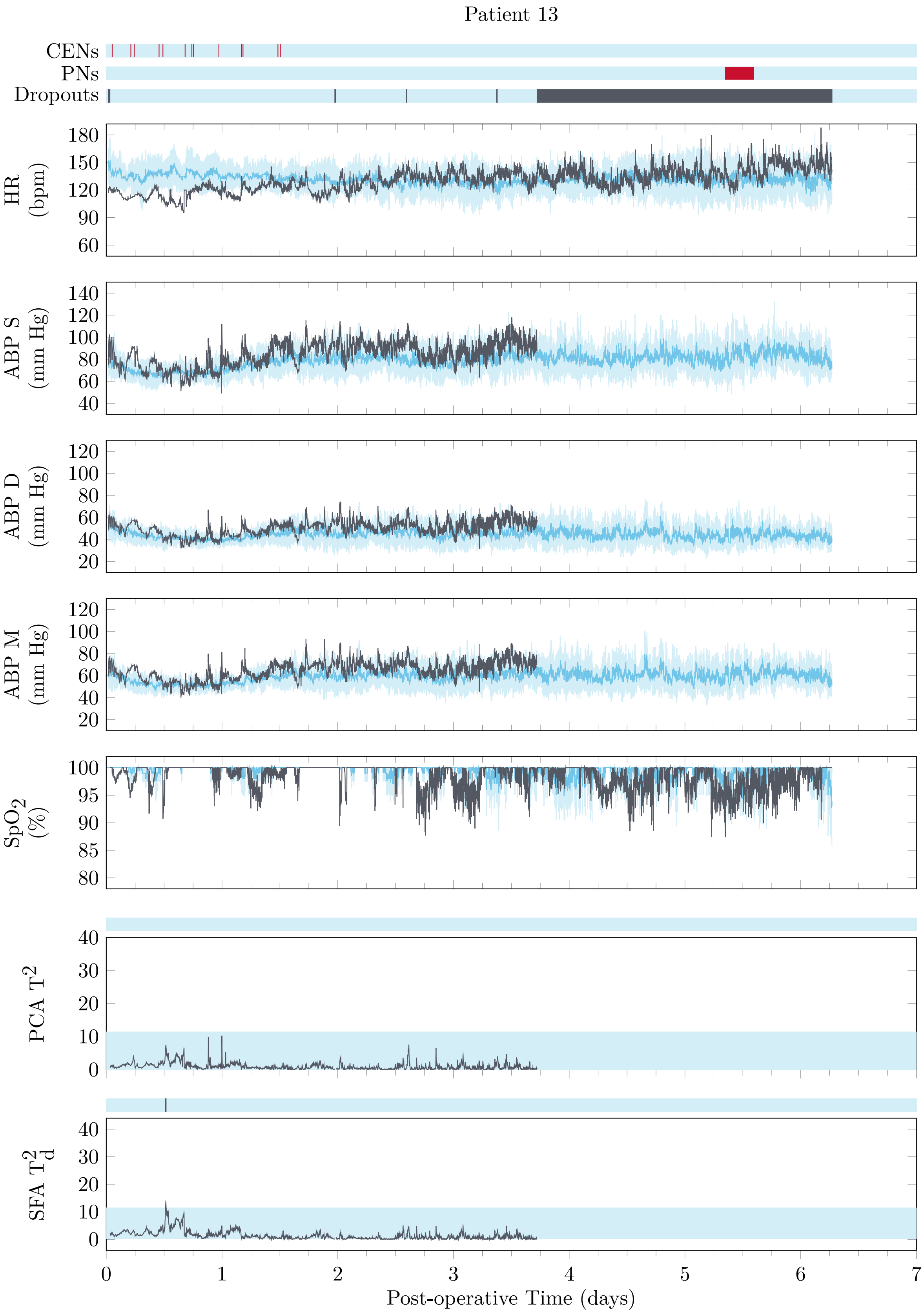

Figure S13: Post-operative monitoring for Patient 13. In each panel, the darker blue line indicates the mean trajectory of the patients other than the current patient and the lighter blue shaded region indicates the region within one standard deviation of this group. The dark grey lines indicate the measurements for the current patient. Spark charts for the clinical event notes, progress notes, and dropouts are also provided.

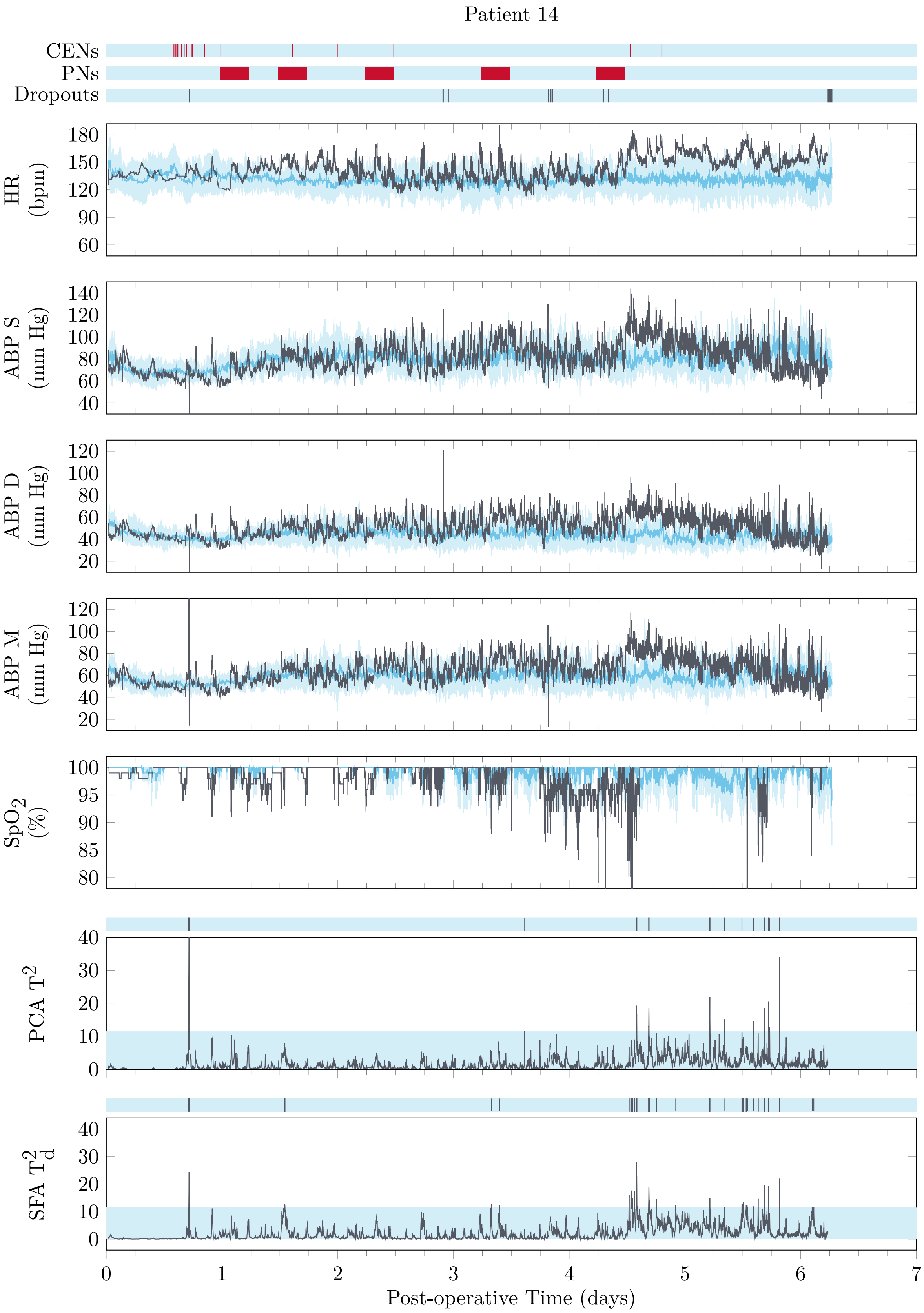

Figure S14: Post-operative monitoring for Patient 14. In each panel, the darker blue line indicates the mean trajectory of the patients other than the current patient and the lighter blue shaded region indicates the region within one standard deviation of this group. The dark grey lines indicate the measurements for the current patient. Spark charts for the clinical event notes, progress notes, and dropouts are also provided.
